# 51130 Risk of Prolonged Opioid Use After Intensive Care Unit Admission

**DOI:** 10.1017/cts.2021.769

**Published:** 2021-03-30

**Authors:** Lia D Delaney, Brooke Kenney, May Hu, Michael Englesbe, Chad Brummett, Jennifer F Waljee

**Affiliations:** 1 University of Michigan Medical School; 2 Center for Healthcare Outcomes and Policy, University of Michigan; 3 Department of Surgery, University of Michigan; 4 Department of Anesthesiology, University of Michigan

## Abstract

ABSTRACT IMPACT: This is the first examination of risk factors for prolonged opioid use after an ICU stay and will inform efforts to strengthen prescribing guidelines and care transition models for patients after critical illness. OBJECTIVES/GOALS: The majority of patients in intensive care units (ICU) receive opioids during admission, and up to 25% receive a prescription at discharge. However, transitions of care and prolonged use after discharge remain unknown. We aim to characterize risk factors for prolonged opioid use after an ICU stay. METHODS/STUDY POPULATION: A retrospective study using insurance claims from Optum Clinformatics ®Data Mart was conducted for opioid-naive adult patients (18-64 years) with an ICU admission from 2010 to 2019. The primary outcome was new persistent opioid use, defined as a continued prescription fill 91-180 days after discharge, in addition to a fill in the first 90 days. The primary exposure was an opioid fill at discharge. The ICU admission was characterized using the Clinical Classification System from the Agency of Healthcare Research and Quality, based on patients’primary diagnosis code. Diagnoses were combined into 11 groups highlighting the affected organ system/mechanism of injury. Logistic regression evaluated the associations of patient demographic and clinical characteristics with the probability of persistent opioid use. RESULTS/ANTICIPATED RESULTS: In this cohort of 90,721 patients discharged from the ICU, 3.3% continued to fill opioids at 6 months. An opioid prescription fill (OR 3.1; 95% CI 28 - 3.3) and benzodiazepine prescription fill (OR 1.6; 95% CI 1.4 - 1.8) within 3 days of ICU discharge were each significantly associated with the development of new persistent opioid use. Patient diagnosis groups of Musculoskeletal/Trauma (OR 2.3; 95% CI 2.0 - 2.6), Neoplasms (OR 1.6; 95% CI 1.5 - 1.9), and GI/Hepatobiliary (OR 1.5; 95% CI 1.3 - 1.8) were significantly more likely to develop new persistent use when compared to the Cardiovascular diagnosis group. DISCUSSION/SIGNIFICANCE OF FINDINGS: Opioid prescriptions at discharge after an ICU stay increase the odds of prolonged opioid use. These results will inform efforts to strengthen prescribing guidelines and care models after a critical illness. Further work will characterize the trajectory of prescribing and patient exposure to high-risk prescribing after ICU discharge.

